# Parechovirus Genotype 3 Outbreak among Infants, New South Wales, Australia, 2013–2014

**DOI:** 10.3201/eid2107.141149

**Published:** 2015-07

**Authors:** Germaine Cumming, Ameneh Khatami, Brendan J. McMullan, Jennie Musto, Kit Leung, Oanh Nguyen, Mark J. Ferson, Georgina Papadakis, Vicky Sheppeard

**Affiliations:** Health Protection New South Wales, Sydney, New South Wales, Australia (G. Cumming, J. Musto, V. Sheppeard);; New South Wales Ministry of Health, Sydney (G. Cumming, K. Leung);; The Children’s Hospital at Westmead, Sydney (A. Khatami);; Sydney Children’s Hospital, Sydney (B.J. McMullan);; University of New South Wales, Sydney (B.J. McMullan, M.J. Ferson);; Western Sydney Local Health District, Sydney (O. Nguyen);; South Eastern Sydney Local Health District, Sydney (M.J. Ferson);; Victorian Infectious Disease Reference Laboratory, Melbourne, Victoria, Australia (G. Papadakis)

**Keywords:** human parechovirus 3, piconarvirus, infantile fever, neonatal infection, sepsis-like syndrome, skin rash, public health surveillance, viruses, New South Wales, Australia

## Abstract

From October 2013 through February 2014, human parechovirus genotype 3 infection was identified in 183 infants in New South Wales, Australia. Of those infants, 57% were male and 95% required hospitalization. Common signs and symptoms were fever >38°C (86%), irritability (80%), tachycardia (68%), and rash (62%). Compared with affected infants in the Northern Hemisphere, infants in New South Wales were slightly older, both sexes were affected more equally, and rash occurred with considerably higher frequency. The New South Wales syndromic surveillance system, which uses near real-time emergency department and ambulance data, was useful for monitoring the outbreak. An alert distributed to clinicians reduced unnecessary hospitalization for patients with suspected sepsis.

## Abstract 2

Syndromic surveillance was useful for outbreak monitoring, and public health response helped reduce hospitalization times.

The clinical manifestations of infection with human parechoviruses (HPeVs), members of the family *Picornaviridiae*, are often indistinguishable from those caused by human enterovirus infections. Over the past decade, outbreaks of human parechovirus genotype 3 (HPeV3) have been reported from the Northern Hemisphere and are particularly well documented in Japan (where the virus was discovered), Canada, the United Kingdom, Denmark, and the Netherlands (*1*–*4*). Of the 16 HPeV genotypes, HPeV3 is the most aggressive and causes a sepsis-like syndrome in neonates (*5*). HPeV infection seems to follow a seasonal pattern; incidence is higher in summer and autumn (*2*,*3*). It can be spread by the fecal–oral and respiratory routes (*4*).

On November 22, 2013, Health Protection New South Wales (NSW), Australia, was notified of a possible cluster of HPeV cases at The Children’s Hospital at Westmead in Sydney. At that time, 7 neonates had experienced rapid onset of acute sepsis-like illness with fever >38°C and a combination of irritability/pain, diarrhea, confluent erythematous rash, tachycardia, tachypnea, encephalitis, myoclonic jerks, and hepatitis. Inquiries revealed that neonates described as “red, hot, angry” had also been admitted to other tertiary children’s hospitals in NSW (*6*). An expert advisory group comprising staff from the NSW Ministry of Health, Health Protection NSW, public health units, and the Sydney Children’s Hospital Network was convened to coordinate the investigation.

On November 25, 2013, PCR detection of HPeV RNA confirmed HPeV infection in 2 of the children. The NSW public health network and clinicians agreed that a surveillance program should be initiated to gather information on the epidemiologic and clinical characteristics and outcomes of children with HPeV infection.

In addition to the public health response, Health Protection NSW issued a media release to alert members of the public to the outbreak. On November 29, 2013, HPeV3 information including a case definition, instructions for accessing diagnostic testing, and recommended clinical management was distributed to all emergency departments, pediatricians, and early childhood health services in NSW. During the outbreak, the expert advisory group met regularly via teleconference to discuss and address any emerging issues. HPeV3 active surveillance activities were concluded on January 31, 2014, while other forms of surveillance continued into February 2014. We describe the epidemiology of the outbreak as observed through several surveillance mechanisms.

## Methods

**Figure 1:**
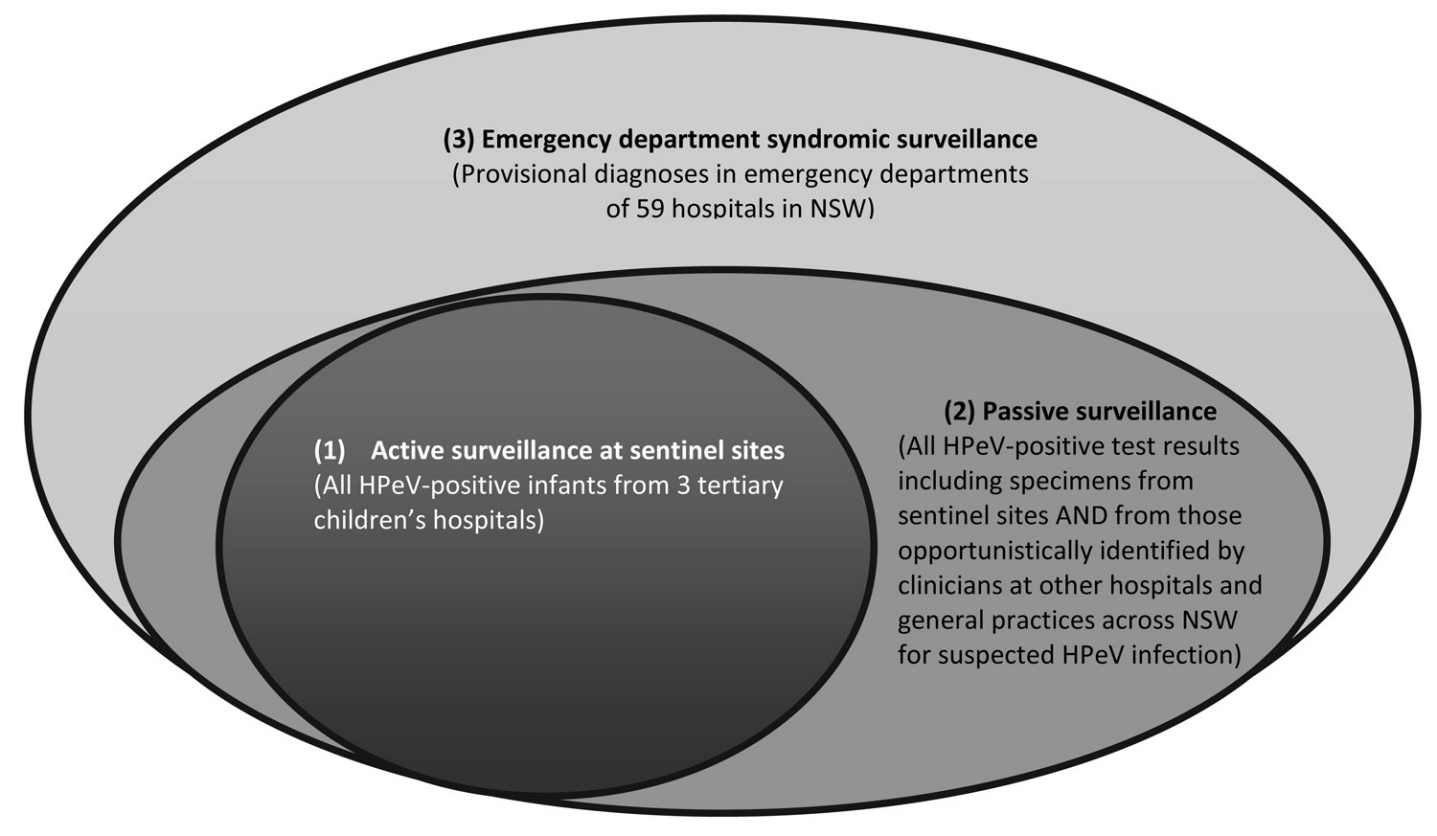
Venn diagram showing capture and overlaps in human parechovirus (HPeV) case identification/reporting resulting from the 3 surveillance mechanisms used during the HPeV outbreak in New South Wales (NSW), Australia, during October 2013–February 2014.

HPeV infection is not a notifiable disease under the Public Health Act 2010 (NSW). This HPeV3 outbreak was detected and reported by clinicians alert to unusual clusters and patterns of disease. Other forms of surveillance were developed as a result of this alert. Surveillance consisted of 3 components: 1) active surveillance (case finding at the sentinel sites); 2) passive surveillance (laboratory reporting of all positive HPeV specimens from sentinel sites and elsewhere in NSW to Health Protection NSW); and 3) syndromic surveillance (reporting of infants seen in emergency departments by the NSW syndromic surveillance system that uses near real-time emergency department and ambulance data [*7*]) (Figure 1). The sentinel sites were 3 tertiary children’s hospitals in NSW: The Children’s Hospital at Westmead, The Sydney Children’s Hospital Randwick, and John Hunter Children’s Hospital Newcastle. Passive and syndromic surveillance continued into February 2014. In addition to surveillance, public health communication in the form of an HPeV information sheet for clinicians was distributed on November 29, 2013, alerting emergency department staff, pediatricians, and early childhood health service staff of current HPeV activity in NSW, providing a description of HPeV infection, and recommending management options (i.e., early laboratory testing and provision of supportive care after receipt of confirmation of HPeV infection).

### Active Surveillance 

Active surveillance activities commenced at the 3 hospitals (sentinel sites) on November 25, 2013, and continued through January 19, 2014; however, some retrospective case finding was included for cases with onset dating back to October 1, 2013, when the outbreak was thought to have started. A patient with a suspected case of HPeV was defined as a neonate or young infant <3 months of age with sepsis-like illness and fever >38.0°C and >2 of the following: irritability/pain, rash, diarrhea, tachycardia, tachypnea, encephalitis, myoclonic jerks, or hepatitis. A patient with a confirmed case of HPeV was a suspected case-patient for whom PCR was positive for HPeV. Clinicians at the sentinel sites collected case information by using an HPeV case investigation form and PCR testing of patient stool, cerebrospinal fluid (CSF), nasopharyngeal aspirate, throat swab, rectal swab, or whole blood samples for HPeV; stool and CSF samples were preferred. (*3*,*8*,*9*). These data were entered into the NSW Notifiable Conditions Information Management System. Weekly reports informed the NSW public health network of outbreak progression.

### Passive Surveillance

All positive HPeV test results from NSW residents referred to the Victorian Infectious Diseases Reference Laboratory (VIDRL) in Melbourne, Victoria (the only laboratory in the region testing for HPeV), from October 1, 2013, through February 2, 2014, were reported to NSW Health. Specimen date; estimated illness onset date; sample type; and patient date of birth, sex, and postcode were recorded in the NSW Notifiable Conditions Information Management System.

### Syndromic Surveillance

The NSW emergency department syndromic surveillance system monitored the number of infants <1 year of age for whom a provisional diagnosis of fever/unspecified infection was made in the emergency department and the number of patients who required hospital admission, including admission to critical care wards. A diagnosis of fever/unspecified infection can include fever symptoms, unspecified viral infection, unspecified viremia, unspecified bacteremia, unspecified bacterial infection, or unspecified infection. Weekly reports compared recent data with historical data from the previous 5 years.

### Laboratory Methods

From all clinical samples, nucleic acid was extracted by using QIAGEN DX reagents (QIAGEN, Hilden, Germany) on a QIAxtractor NA extraction robot (QIAGEN). cDNA was synthesized by using a method previously described (*10*) and was tested in an HPeV real-time PCR selective for the 5′ untranslated region, which was developed at VIDRL (*10*). (The primer and probe sequence details for this assay can be supplied upon request to G.C.)

Molecular analysis to obtain the HPeV genotype was performed on selected samples that had been positive by real-time PCR. Specimens from 41 patients were selected for genotyping on the basis of ensuring representation of infants’ geographic locations, ages, sex, illness onset dates, specimen types, and sex. Identification of specific HPeV genotypes was obtained through amplification of the viral protein 1 gene by use of a gel-based seminested PCR (*11*). The generated PCR products were sequenced and compared with reference sequences by using the primers and methods described elsewhere (*12*).

### Statistical Analyses

Descriptive analysis of epidemiologic variables and patient demographic characteristics were performed. Characteristics of infants <3 months of age, such as length of stay (LOS), were compared by using *t*-tests to determine the effects of public health messaging. Analyses was performed by using SAS version 9.2 (SAS Institute, Inc., Cary, NC, USA) and Microsoft Excel (Microsoft, Redmond, WA, USA).

## Results

### Laboratory Findings

From November 1, 2013, through February 28, 2014, a total of 420 specimens were submitted for HPeV PCR testing; for some patients, >1 specimen was submitted. PCR results were HPeV positive for 289 (69%) specimens from 198 patients (Table 1). In addition to confirming HPeV RNA in samples from 198 patients in NSW, HPeV type 3 was identified from all 41 (21%) positive samples for which molecular analysis was subsequently performed. The phylogenetic tree demonstrating all HPeV3 isolates genotyped at VIDRL during the outbreak is reported elsewhere (*6*).

**Table 1:** Results of PCR testing of specimens from patients from New South Wales received at the VIDRL, Melbourne, Victoria, Australia, November 1, 2013–February 28, 2014 *

Test	Specimens, no. (%)	Patients, no. (%)
Total no.	420 (100)	308 (100)
PCR+ for HPeV	289 (69)	198 (64)
PCR+ for HPeV only	285 (68)	194 (63)
PCR+ for HPeV and EV†	4 (1)	4 (1)
PCR+ for EV only	15 (4)	14 (5)
PCR+ for EV	19 (5)	18 (6)
PCR− for HPeV	131 (31)	110 (36)
PCR− for HPeV and EV	116 (28)	96 (31)

*EV, enterovirus; HPeV, human parechovirus;VIDRL, Victorian Infectious Disease Reference Laboratory: +, positive; –, negative. †Because of how the testing algorithm is set up at VIDRL, all samples tested for HPeV are also tested for EV. EV results are shown to demonstrate the small amount of EV detected in this HPeV outbreak period, with even fewer co-infections.

**Figure 2:**
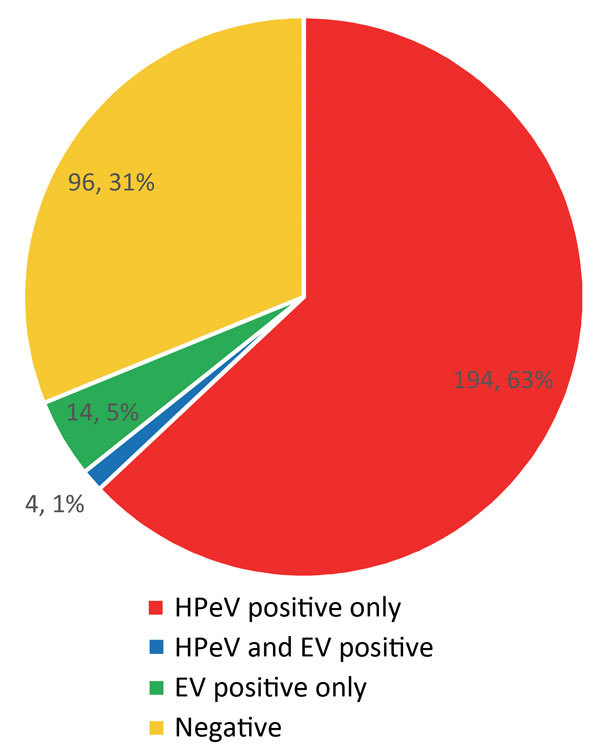
Patient human parechovirus (HPeV) and enterovirus (EV) results for all 198 patients in New South Wales, Australia, tested by the Victorian Infectious Diseases Reference Laboratory during November 1, 2013–February 28, 2014.

Because of the algorithms used in the testing, enterovirus results were also available for all samples submitted (Table 1). A total of 194 patients had HPeV infection only, 4 had dual infections (HPeV and enterovirus), and 14 had enterovirus infection only (Figure 2). Results for the rest of the patients were negative. Focusing on CSF and fecal samples, 123 (73%) of 168 CSF samples were HPeV positive by PCR (mean cycle threshold [C_t_] detection value 31.6), and 114 (73%) of 156 fecal samples were positive (mean C_t_ 27.2) (Table 2). PCR was run for 45 cycles; therefore, C_t_ values >45 were considered negative.

**Table 2:** HPeV testing of specimens from patients from New South Wales, received at the VIDRL, Melbourne, Victoria, Australia, November 1, 2013–February 28, 2014, by specimen type*

Sample and result	Specimens, no. (%)	Patients, no. (%)
Total	420 (100)	308 (100)
CSF		
Total samples tested	168 (40)	161 (52)
HPeV+ (mean C_t_ 31.6)†	123 (73)	116 (72)
PCR+ for HPeV and EV	1 (1)	1 (1)
Stool		
Total samples tested	156 (37)	147 (48)
HPeV+ (mean C_t_ 27.2)	114 (73)	106 (72)
PCR+ for HPeV and EV	2 (1)	2 (1)
Other		
Total samples tested	96 (23)	8 (28)
HPeV+	52 (54)	45 (52)
PCR+ for HPeV and EV	1 (1)	1 (1)
Sequenced: HPeV3	41 (14)	41 (21)

*CSF, cerebrospinal fluid; C_t_, cycle threshold; EV, enterovirus; HPeV, human parechovirus; VIDRL, Victorian Infectious Disease Reference Laboratory: +, positive. †At VIDRL, PCRs are run for 45 cycles, so any C_t_ value greater than 45 is considered negative.

### Active and Passive Surveillance Findings

**Figure 3:**
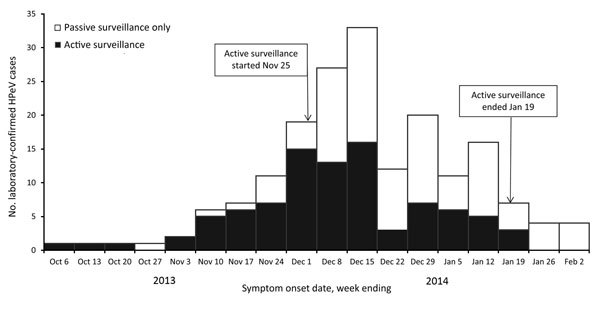
Number of laboratory-confirmed human parechovirus (HPeV) cases identified by active and passive surveillance, by week of symptom onset, in New South Wales (NSW), Australia, during the October 2013–early February 2014 outbreak (total = 183 cases). Source: NSW Notifiable Conditions Information Management System data (http://www.health.nsw.au/epidemiology/Pages/Notifiable-diseases.aspx), February 18, 2014. Source: NSW Notifiable Conditions Information Management System data (http://www.health.nsw.au/epidemiology/Pages/Notifiable-diseases.aspx), February 18, 2014.

Active surveillance identified 94 infants whose illness met the definition of a confirmed case (patient <3 months of age and HPeV-positive laboratory results, originating from sentinel sites). Passive surveillance spanning specimen collection dates from October 1, 2013, through February 2, 2014, identified another 89 laboratory-confirmed cases in NSW in patients 0–17 months of age. The outbreak peaked during the first 2 weeks of December 2013 (Figure 3).

**Figure 4:**
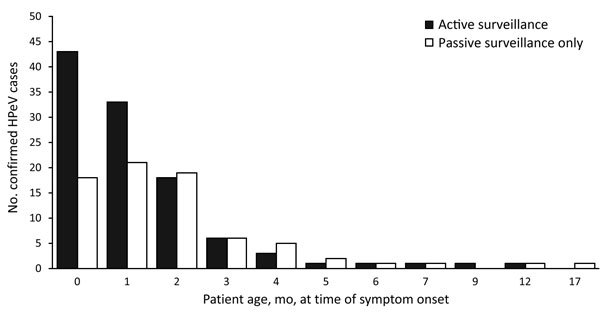
Age distribution of 183 patients with confirmed human parechovirus (HPeV) infection at time of symptom onset, detected by active and passive surveillance, New South Wales (NSW), Australia, October 1, 2013–February 2, 2014. Source: NSW Notifiable Conditions Information Management System data (http://www.health.nsw.au/epidemiology/Pages/Notifiable-diseases.aspx), February 18, 2014.

More cases (105 [57%]) were in male than female patients; median patient age was 1.51 months (or median 46 days, range 0–537 days) (Figure 4; Table 3). Intrafamily HPeV3 transmission was identified in twins, 2 parent–child pairs, and a set of cousins. A descriptive case series of the infants infected with HPeV3 during this outbreak, containing further clinical details on select cases, is reported elsewhere (*6*).

**Table 3:** Characteristics of patients with laboratory-confirmed HPeV detected by active surveillance, New South Wales, Australia, October 1, 2013–February 2, 2014*

Characteristic	Passive surveillance for all laboratory-confirmed HPeV case-patients,† no. (%)	Active surveillance, enhanced data for all HPeV case-patients from sentinel sites,‡ no. (%), n = 108

<3 mo	>3 mo	All ages
Total	183 (100)	94 (87)	14 (13)	108 (100)
Sex				
F	78 (43)	45 (48)	6 (43)	51 (47)
M	105 (57)	49 (52)	8 (57)	57 (53)
Admitted to ward	NA	89 (95)	14 (100)	103 (95)
Length of stay, days				
Minimum	NA	1	1	1
Maximum	NA	13	11	13
Mean	NA	4.5	3.7	4.4
Admitted to ICU	NA	28 (30)	2 (14)	30 (28)
Clinical features				
Fever	NA	81 (86)	9 (64)	90 (83)
Irritability/pain	NA	75 (80)	11 (79)	86 (80)
Diarrhea/loose stool	NA	29 (31)	2 (14)	31 (29)
Tachypnea	NA	26 (28)	3 (21)	29 (27)
Tachycardia	NA	64 (68)	7 (50)	71 (66)
Hepatitis	NA	9 (10)	1 (7)	10 (9)
Rash	NA	58 (62)	9 (64)	67 (62)
Encephalitis	NA	7 (7)	0 (0)	7 (6)
Myoclonic jerks	NA	4 (4)	1 (7)	5 (5)

*Table source: NSW Notifiable Conditions Information Management System data (http://www.health.nsw.au/epidemiology/Pages/Notifiable-diseases.aspx), February 18, 2014. HPeV, human parechovirus; ICU, intensive care unit; NA, not available; NSW, New South Wales; VIDRL, Victorian Infectious Disease Reference Laboratory, Melbourne, Victoria, Australia. †Passive laboratory surveillance ceased at NSW Health on February 2, 2014, leaving at a total of 183 laboratory-confirmed HPeV cases. This number differs from the total of 198 confirmed HPeV cases reported by VIDRL surveillance, which continued through to February 28, 2014, and identified another 15 cases. ‡Sentinel surveillance sites collected additional demographic, hospital, and clinical data onto the HPeV case investigation forms, which were entered into the NSW Notifiable Conditions Information Management System. No information on hospital stay and clinical features was available for cases identified by laboratory-only surveillance. Surveillance at sentinel sites is a subset (59%) of the outbreak total of 183 laboratory-confirmed cases.

Analysis of case investigation forms from the sentinel sites reporting HPeV signs and symptoms showed that the most commonly reported signs for infants <3 months of age were fever (86%), irritability/pain (80%), tachycardia (68%), and rash (62%) (Table 3). Similar signs were displayed by those >3 months of age; however, 20% fewer in this age group had fever and tachycardia (Table 3). As described previously, all infants at the sentinel sites were well at the time of hospital discharge, and further longitudinal follow-up studies are examining the long-term outcomes of these infections having occurred in early life (*6*).

Of the 183 confirmed cases, 108 (59%) were captured by the 3 sentinel surveillance sites, and another 75 (41%) were diagnosed at other hospitals. Most (57%) patients resided in the Sydney metropolitan area, and the remaining 43% were from regional or rural areas of NSW. This finding compares with 64% and 36% of the NSW population residing in metropolitan Sydney and regional/rural areas, respectively (*13*).

Analysis of case investigation forms for the 108 patients at the sentinel sites also showed that 103 (95%) patients were admitted to hospital and had an average LOS of 4.4 (1–13) days (Table 3). Mean LOS was greater for infants <3 months of age (4.5, range 1–13 days) than for those >3 months of age (3.7, range 1–11 days). The rate of admission to an intensive care unit was lower for older infants (14%) than for those <3 months of age (30%) (Table 3).

**Figure 5:**
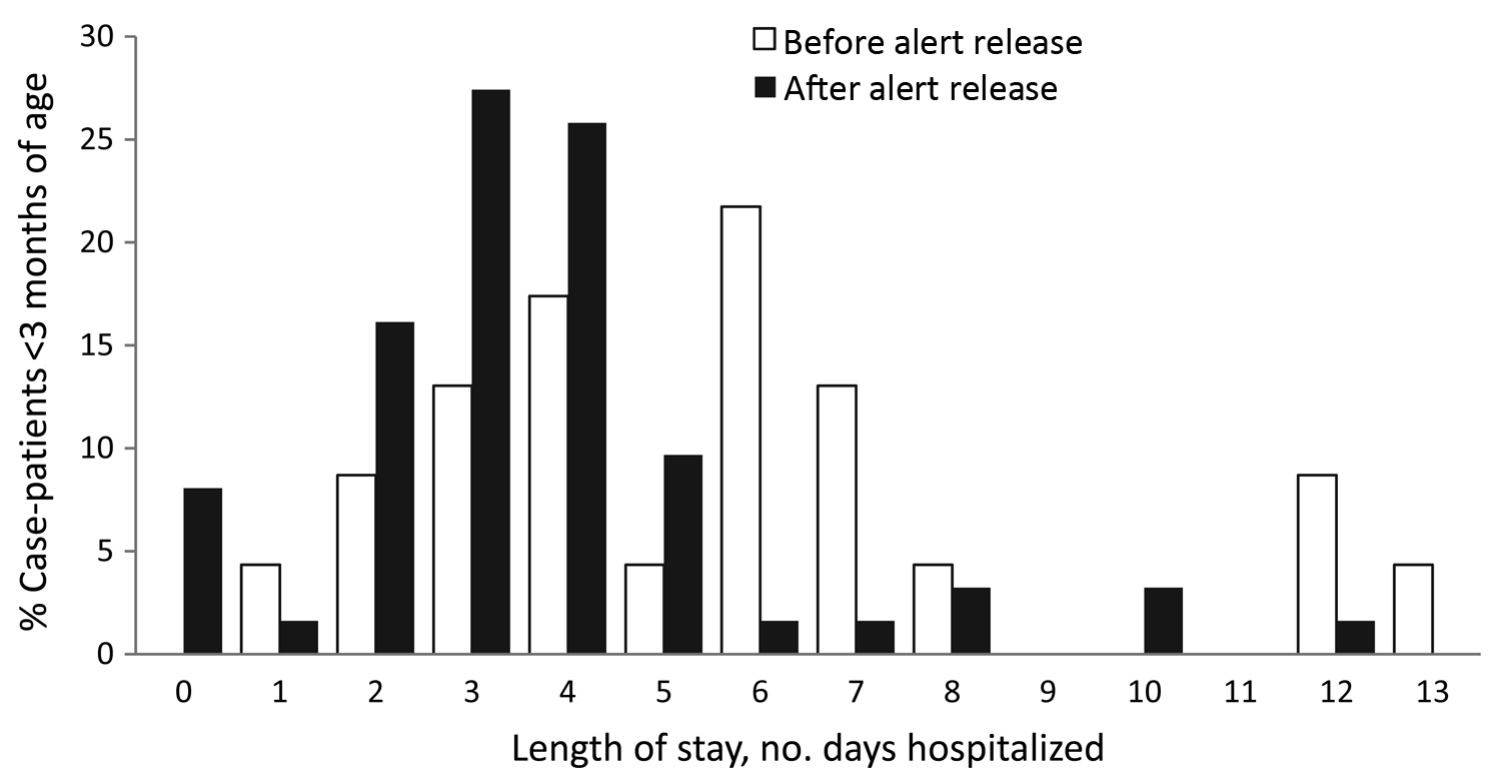
Distribution of hospital length of stay for infants <3 months of age at sentinel sites during active surveillance of the human parechovirus outbreak in New South Wales (NSW), Australia, October 1, 2013–February 2, 2014. Source: NSW Notifiable Conditions Information Management System data (http://www.health.nsw.au/epidemiology/Pages/Notifiable-diseases.aspx), February 18, 2014.

The trend statistic on the distribution of LOS in infants <3 months of age showed that LOS was significantly reduced among infants <3 months of age who became ill after the HPeV alert was sent on November 29, 2013, to emergency departments and pediatricians. Mean LOS at the sentinel sites was 5.7 days before and 4.0 days after sending of the alert (Satterthwaite *t*-test, p<0.05) (Figure 5).

### Syndromic Surveillance Findings

**Figure 6:**
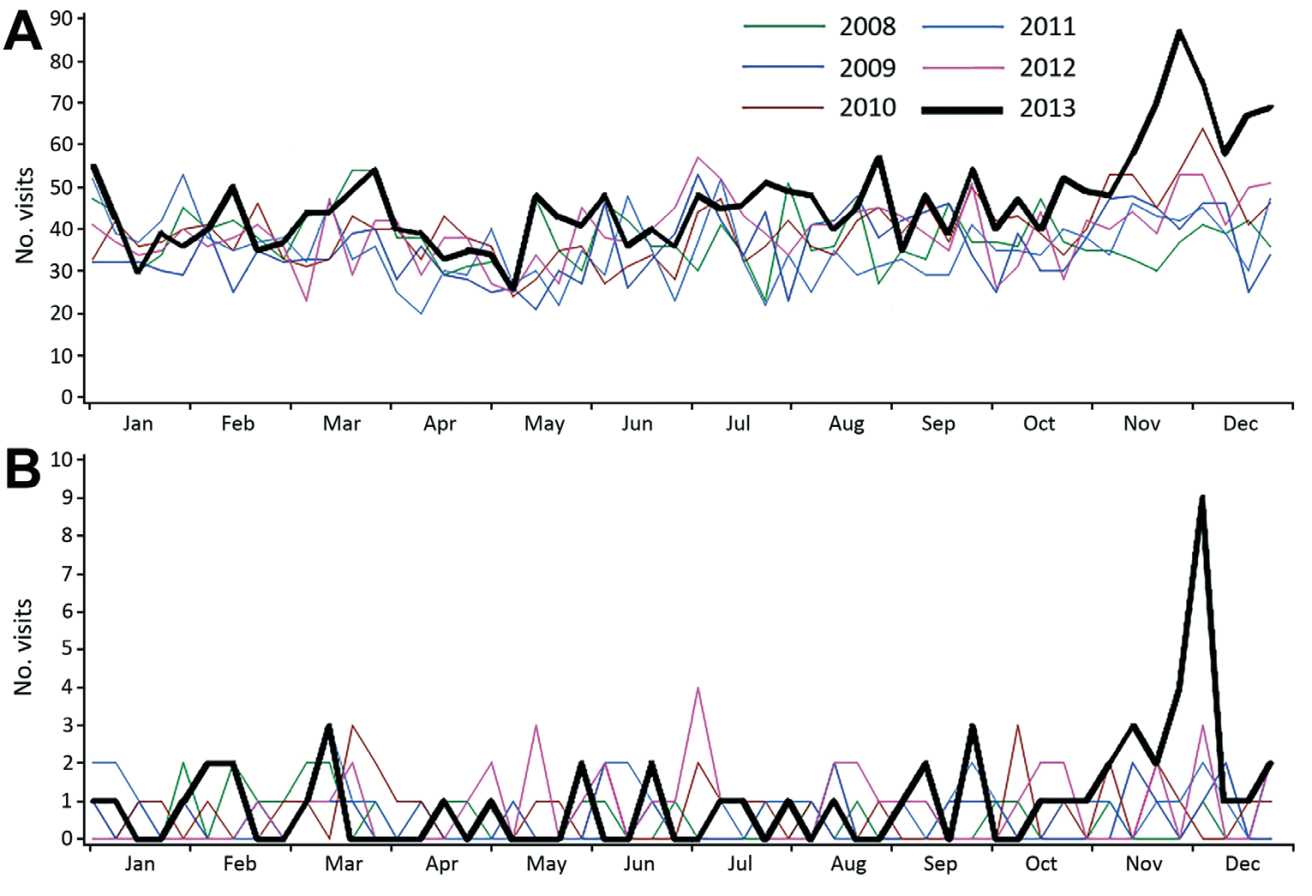
Total weekly counts of visits to the emergency department for fever or unspecified infection for which patients were (A) admitted to ward and (B) admitted to critical care for 2013, compared with each of the 5 previous years, children <1 year of age, for 59 hospitals in New South Wales, Australia. Source: Emergency department syndromic surveillance report produced on June 3, 2014.

The number of infants <1 year of age with a provisional emergency department diagnosis of fever/unspecified requiring hospital admission began to rise sharply in mid-November 2013 and peaked during the first week of December (Figure 6, panel A). At the peak, the number of admissions was 83, compared with an average of 52 for the same week in previous years. Admissions remained elevated until mid-January 2014, when they returned to background levels. Admissions to critical care wards spiked during the second week of December, when 9 patients were admitted, well above the average of 1 admission per week for the same week in previous years (Figure 6, panel B). During the surveillance period, most admissions were to the 2 children’s hospitals in metropolitan Sydney. 

## Discussion

This outbreak is probably one of the first large parechovirus outbreaks to be reported in Australia. We observed a large number of cases over a relatively short period (≈4 months), peaking around late spring/early summer, which is earlier than documented seasonality for parechovirus in the Northern Hemisphere (*3*). Although sequencing in this series was incomplete (21% of patients), this parechovirus outbreak was determined to have been caused by HPeV3 for the following reasons: all sequenced HPeV-positive samples were genotype 3; the epidemiology and spectrum of illness seen by clinicians at the sentinel sites was relatively homogenous and in keeping with other reports of HPeV3 infections in infants (*3*,*14*); and PCR was enterovirus positive for only 18 patients but HPeV positive for 198 patients.

Infected infants in this outbreak were older than those reported elsewhere. The median age of 46 days was higher than that reported in Denmark (39 days) and the Netherlands (40 days) (*3*,*14*). The occurrence of a substantial proportion (17%) of HPeV3 infections in infants >3 months of age was consistent with results from a US study reporting 18% of cases in infants >60 days of age but contrary to other data indicating that HPeV3 infection occurs almost exclusively in infants <3 months of age (*2*,*4*).

The age cutoff in the case definition for active surveillance at the sentinel sites may have introduced a bias toward HPeV infection being more frequently suspected in infants <3 months of age; thus, these infants might have undergone more HPeV testing than older infants, thereby underestimating the true mean age of infants affected in this outbreak. Had the age cutoff in the case definition been set at 12 months (all infants), bias toward younger infants would have been avoided, and the median age might have been older than that reported here.

After the November 29, 2013, release of the HPeV alert to emergency department staff and pediatricians, mean LOS among infants <3 months of age decreased by 30%, from ≈5.7 days at the start of the outbreak to 4.0 days (p = 0.0250). This statistically significant finding reflects a degree of effectiveness of public health messaging in raising clinician awareness of HPeV. During the latter part of the outbreak, after Health Protection NSW issued the alert, informed clinicians may have felt more comfortable discontinuing treatment and discharging infants sooner if their illness met the criteria of the HPeV case definition. The alert seemed to result in improved clinical management of cases, avoidance of unnecessary prolonged exposure to empirically prescribed antimicrobial drugs, provision of appropriate supportive treatment, and earlier discharge from hospital. The mean LOS during the NSW outbreak (4.4 days) was lower than that reported in the Netherlands (7 days), according to an analysis of retrospective diagnoses when no public health intervention would have taken place (*8*). Another factor that could contribute to reduced LOS in the latter part of an outbreak is the increasing age of young infants becoming ill with HPeV infection in the second half of the outbreak, although this factor was not statistically significant in this dataset (*15*).

The male:female split in this outbreak was less pronounced than that reported for other studies, although more cases consistently occurred in boys. Others have reported a higher preponderance of infection in boys, ranging from 70% to 90%, compared with our finding of 57% (*2*,*5*,*8*,*14*).

Clinical signs reported in the literature for HPeV3-infected infants were consistent with our findings of fever, irritability, and encephalitis (*2*,*6*,*8*). However, rash occurred with much higher frequency (62% vs. 17%) in the NSW outbreak than in other outbreaks (*8*).

Syndromic surveillance that used emergency department data proved to be a useful and timely way to monitor emergency department hospital admissions temporally associated with the HPeV3 outbreak. Increased presentation of infants <1 year of age with a provisional emergency department diagnosis of fever/unspecified infection requiring hospital admission, in particular admission to critical care, were associated with increased detection of HPeV3 at the clinical level. Emergency department syndromic surveillance reports also helped confirm an overall decline in admissions from emergency departments in early 2014, supporting the eventual withdrawal of active surveillance. Emergency department syndromic surveillance in NSW does not routinely monitor age-specific or admission-specific (hospital ward or critical care unit) aberrations; that is, all children <5 years of age are monitored as a group. In the future, data generated through the emergency department syndromic surveillance system may continue to be useful for monitoring the evolution of an HPeV or similar outbreak. The cost of maintaining emergency department syndromic surveillance like that used during this outbreak (fever/unspecified infection among children <1 year of age and admission to hospital) would need to be considered. Costs of doing so include personnel time for checking reports and investigating signals, infrastructure costs, and opportunity costs; choosing to monitor HPeV3-related signals indirectly means that signals for diseases that are not prioritized are not monitored. In addition, the sensitivity of this grouping will need to be tested in future outbreaks before it can be considered a reliable proxy indicator of a seasonal outbreak

We initiated sentinel surveillance on the assumption that nearly all neonates with severe HPeV3 disease would be referred to 1 of the 3 tertiary children’s hospitals in NSW. Through passive surveillance we identified an additional 41% of HPeV patients who had been seen at other health facilities. Enhanced data were not collected for these presumably milder cases, which has limited our capacity to conduct more extensive significance testing across the observed differences in clinical features. Recording more information on potential exposures (e.g., infants’ daycare attendance, existence of older siblings, and occurrence of family illness in weeks preceding infants’ admission) would have further aided our understanding of HPeV3 transmission in the community.

## Conclusion

The objectives of HPeV surveillance were achieved: document the outbreak, describe the clinical features of cases, help inform clinicians and the public, monitor the evolution of the outbreak, and add to the knowledge base. The HPeV3 infection outbreak in NSW, Australia, differed slightly from that documented in the Northern Hemisphere; the NSW outbreak apparently affected slightly older infants (as well as neonates and young infants), cases were more evenly split between boys and girls, and rash occurred at a considerably higher frequency. The value of awareness-raising communication strategies was demonstrated by the statistically significant 30% reduction in LOS during the outbreak immediately after release of the alert to emergency department staff and pediatricians. This alert helped to minimize unneeded use of antimicrobial drugs and reduce unnecessary hospitalization. Although active surveillance is resource intensive, it has helped to define HPeV3 infection in NSW and link it with a syndromic surveillance indicator in the emergency department syndromic surveillance system. Syndromic surveillance is a potentially useful proxy indicator that should be considered for future detection and surveillance of seasonal outbreaks of viral infections. 

## References

[1] Selvarangan R, Nzabi M, Selvaraju SB, Ketter P, Carpenter C, Harrison CJ. Human parechovirus 3 causing sepsis-like illness in children from Midwestern United States. Pediatr Infect Dis J. 2011;30:238–42. 10.1097/INF.0b013e3181fbefc8 20948454

[2] Sharp J, Harrison CJ, Puckett K, Selvaraju SB, Penaranda S, Nix WA, Characteristics of young infants in whom human parechovirus, enterovirus or neither were detected in cerebrospinal fluid during sepsis evaluations. Pediatr Infect Dis J. 2013;32:213–6 . 23042051 10.1097/INF.0b013e318276b328 PMC4637937

[3] Fischer TK, Midgley S, Dalgaard C, Nielsen AY. Human parechovirus infection, Denmark. Emerg Infect Dis. 2014;20:83–7. 10.3201/eid2001.130569 24377661 PMC3884717

[4] Harvala H, Wolthers KC, Simmonds P. Parechovirus in children: understanding a new infection. Curr Opin Infect Dis. 2010;23:224–30. 10.1097/QCO.0b013e32833890ca 20414971

[5] Guo Y, Duan Z, Qian Y. Changes in human parechovirus profiles in hospitalised children with acute gastroenteritis after a three-year interval in Lanzhou, China. PLoS ONE. 2013;8:e68321. 10.1371/journal.pone.0068321 23844186 PMC3700866

[6] Khatami A, McMullan B, Webber M, Stewart P, Francis S, Timmers K, Sepsis-like disease in infants due to human parechovirus type 3 during an outbreak in Australia. Clin Infect Dis. 2015;60:228–36. 10.1093/cid/ciu784 25301212

[7] Muscatello DJ, Churches T, Kaldor J, Zheng W, Chiu C, Correll P, An automated, broad-based, near real-time public health surveillance system using presentations to hospital emergency departments in New South Wales, Australia. BMC Public Health. 2005;5:141. 10.1186/1471-2458-5-141 16372902 PMC1361771

[8] Wolthers KC, Benschop KS, Schinkel J, Molenkamp R, Bergevoet RM, Spijkerman IJ, Human parechovirus as an important viral cause of sepsis-like illness and meningitis in young children. Clin Infect Dis. 2008;47:358–63. 10.1086/589752 18558876

[9] Shoji K, Komuro H, Miyata I, Miyairi I, Saitoh A. Dermatologic manifestations of human parechovirus type 3 infection in neonates and infants. Pediatr Infect Dis J. 2013;32:233–6 . 23190775 10.1097/INF.0b013e31827b1fd0

[10] Druce J, Tran T, Kelly H, Kaye M, Chibo D, Kostecki R, Laboratory diagnosis and surveillance of human respiratory viruses by PCR in Victoria, Australia, 2002–2003. J Med Virol. 2005;75:122–9. 10.1002/jmv.20246 15543580 PMC7166941

[11] Nix WA, Maher K, Pallansch MA, Oberste MS. Parechovirus typing in clinical specimens by nested or semi-nested PCR coupled with sequencing. J Clin Virol. 2010;48:202–7. 10.1016/j.jcv.2010.04.007 20472496

[12] Papadakis G, Chibo D, Druce J, Catton M, Birch C. Detection and genotyping of enteroviruses in cerebrospinal fluid in patients in Victoria, Australia, 2007–2013. J Med Virol. 2014;86:1609–13 . 10.1002/jmv.23885 24474149

[13] Australian Bureau of Statistics. Estimated resident population (ERP) by region, age, and sex, 2001 to 2013 [cited 2014 Feb 24]. http://www.abs.gov.au/.

[14] Benschop KS, Schinkel J, Minnaar RP, Pajkrt D, Spanjerberg L, Kraakman HC, Human parechovirus infections in Dutch children and the association between serotype and disease severity. Clin Infect Dis. 2006;42:204–10. 10.1086/498905 16355330

[15] Lenski RE, May RM. The evolution of virulence in parasites and pathogens: reconciliation between two competing hypotheses. J Theor Biol. 1994;169:253–65. 10.1006/jtbi.1994.1146 7967617

